# Polymeric Systems of Antimicrobial Peptides—Strategies and Potential Applications

**DOI:** 10.3390/molecules181114122

**Published:** 2013-11-14

**Authors:** Marcin Sobczak, Cezary Dębek, Ewa Olędzka, Ryszard Kozłowski

**Affiliations:** 1 Department of Inorganic and Analytical Chemistry, Faculty of Pharmacy, Medical University of Warsaw, ul. Banacha 1, Warsaw 02-097, Poland; E-Mail: eoledzka@wp.pl; 2 Institute for Engineering of Polymer Materials and Dyes, ul. Marii Skłodowskiej-Curie 55, Toruń 87-100, Poland; E-Mails: c.debek@ipgum.pl (C.D.); r.kozlowski@impib.pl (R.K.)

**Keywords:** biomedical polymers, peptides with antimicrobial activity, polymeric carriers, biodegradable polymers

## Abstract

The past decade has seen growing interest in the investigation of peptides with antimicrobial activity (AMPs). One approach utilized in infection control is incorporation of antimicrobial agents conjugated with the polymers. This review presents the recent developments on polymeric AMP carriers and their potential applications in the biomedical and pharmaceutical fields.

## 1. Introduction

In the past years, technology of natural, semi- and synthetic polymeric materials with antimicrobial activity represents one of the most rapidly advancing areas in pharmacy, medicine and biomedicine. According to WHO data, the number of deaths caused by infectious illnesses is still increasing. Generally, infectious diseases are responsible for deaths of more people than any other single cause. The infections are usually triggered by bacteria, viruses, fungi, and protozoa. Polymeric materials with antimicrobial activity are used in many fields, particularly in medical device technology, drug delivery systems, health care products, hygienic applications, textiles, water purification systems, food packaging, *etc.* [1,2,3,4,5,6,7,8].

One of the most problematic issues faced by modern medicine are resistant microorganisms that mutate rapidly and easily, making their elimination difficult. Antibiotic-resistant bacteria are an important threat to public health due to the slow development of new antibiotics to replace those that become ineffective [1,2,3,4,5,6,7,8]. Therefore, polymer or bioorganic chemists are still actively involved in designing and obtaining new effective drugs with antimicrobial activity and biocides or drug delivery systems containing those active substances. One particular approach towards an improved use of drugs and biocides for therapeutic applications is use of polymeric carriers or polymer-active substance conjugate systems. The polymeric carriers and macromolecular conjugates of substances with antimicrobial activity exhibit unique pharmacokinetics, distribution, and pharmacological efficacy [9].

There are currently two main methods for obtaining polymeric systems with antimicrobial substances (AS). The first method consists of using AS-immobilized polymeric matrices. AS can be immobilized via incorporation into a variety of polymeric materials or adsorption onto a variety of surfaces where they still retain their ability to bind and kill germs [10]. The rate of AS release depends on the hydrophilic-hydrophobic properties, molecular weight and polydispersity of the polymer as well as the process conditions (pH, temperature, presence of enzymes, *etc.*) [9]. The second method involves linking of an AS onto a polymer via covalent bonds. There are several types of labile bonds (e.g., carbonate, ester, urethane, orthoester, amide, ether, anhydride) that can be used to form biodegradable or bioresorbable polymeric AS conjugates [9].

The most promising group of substances which could be used in AS carrier technology are biodegradable and bioresorbable polymers. These polymers are considered to be very attractive because they can undergo hydrolysis to produce non-toxic compounds metabolized *in vivo* and in the environment [9]. Moreover, they exhibit unique kinetics of AS release and distribution and efficacy.

A lot of biodegradable or bioresorbable natural (e.g., alginic acid, chitosan, collagen, fibrin, gelatin, hyaluronic acid, polyhydroxyalkanoates, starch), semi-synthetic (e.g., pulp modified derivatives, cyclodextrins) and synthetic polymers (e.g., aliphatic polyesters or polycarbonates, aliphatic polyurethanes, polyorthoesters, poly(ethylene oxide), copolymers of cyclic esters and carbonates) are used as AS carriers [9,10,11,12,13,14,15,16,17]. Synthetic biodegradable polymers appear to be a highly attractive option due to their versatility, flexibility and molecular parameters compared to natural biodegradable biopolymers or their modified derivatives. Polyesters (e.g., polylactide (PLA), polyglycolide (PGA), poly(ε-caprolactone) (PCL) and poly(γ-valerolactone) (PVL), or copolymers of *rac*-lactide, L-lactide, ε-caprolactone and glycolide) are representatives of a family of polymers widely utilized in AS delivery systems due to their biodegradability and biocompatibility features [9,10,11,12,13,14,15,16,17].

The antimicrobial peptides (AMPs) are an important group of antimicrobial substances. The term “AMPs” is used to denote a large number of small proteins that can kill or inhibit growth of various microorganisms. Many different AMPs from various families have been discovered in non-vertebrates and vertebrates. They are characterized by a broad spectrum of antimicrobial activity against bacteria (both Gram-positive and Gram-negative ones), viruses, and fungi [18,19,20,21,22,23]. AMPs are characterized by their small size (12–50 amino acids), the arginine and lysine residues responsible for their positive charge, and an amphipathic structure that enables them to interact with microbial membranes [18]. There are several hypotheses describing the mechanism of antimicrobial, antiviral or antifungal activity of AMPs [18]. Four distinct groups of AMPs have been discovered: β-sheet peptides stabilized by two to four disulfide bridges (e.g., human α- and β-defensins, plectasin or protegrins), α-helical peptides (e.g., LL-37, cecropins or magainins), extended structures rich in glycine, proline, tryptophan, arginine or histidine (e.g., indolicidin), and loop peptides with one or disulfide bridge (e.g., bacteriocins) [24,25]. AMPs can be synthesized nonribosomally (e.g., gramicidins, polymyxins, bacitracins, glycopeptides) or ribosomally [24]. The practical applications of AMPs are highly interesting. About 1,000 natural AMPs may serve as lead compounds in future development although currently only a few AMPs are approved for clinical use [18,26].

Peptidomimetic systems with antimicrobial activity are interesting and perspective promising groups of antimicrobial materials. AMP mimetics are constructed from peptoids, β-peptides, arylamides, oligomers, or phenylene ethynylenes. These substances are designed to capture the central physicochemical features of their natural AMP archetypes thereby mimicking peptide activity and function [20,27,28,29,30]. Peptoids are active against e.g., against *Mycobacterium bovis* bacille Calmette–Guérin (BCG), *Mycobacterium tuberculosis*, *Pseudomonas aeruginosa* biofilms [31,32,33]. Oligo-acyl-lysine derivatives are active *in vitro* and *in vivo* against Gram-negative bacteria, with no haemolytic activity [34]. Phenylalkyne and arylamide compounds express antibacterial, antifungal, and anti-inflammatory activities [35,36]. A number of recent review papers have been devoted to describing the potential and mechanisms of action of peptidomimetic systems [10,37,38].

Recently, many studies have focused on AMPs and AMP polymeric systems due to their unique properties. In the coming years, the number of AMPs’ applications will certainly increase. The new developments will probably concern AMP delivery systems (mainly transdermal therapeutic systems) [39,40,41,42]. Many peptides are already used in medicine, e.g., daptomycin (Cubicin^®^, Cubist Pharmaceuticals, Lexington, MA, USA), dermicydin 1 (University of Eberharda Karola, Tübingen, Germany), gramicidin (many manufacturers), human β-defensin 3 (Harvard Medical School, Boston, MA, USA), lactoferrin (Pet King Brands, Westmont, IL, USA), lysostaphin (Biosynexus, Gaithersburg, MD, USA), lizozyme (Neova Technologies Inc, Abbotsford, BC, Canada), Nisin A (Biosynexus), pexiganan (Genaera Corporation, New Hope, PA, USA), plectasin (Novozymes, Bagsværd, Denmark), polymyxin E (many manufacturers), psoriazyna (Christiana-Albrechta University, Kiel, Germany), P-113 (Demegen, Pittsburgh, PA, USA). However, there are a lot of new antimicrobial peptides displaying interesting properties, which are currently under development, such as plectasin NZ2114. It has yet to enter clinical development, but pre-clinical studies suggest that it possesses potent bactericidal activity against Gram-positive pathogens.

As the therapeutic efficacy and safety of ASs administered by conventional methods is regrettably limited, drug delivery systems (also AMPs) have been a centre of considerable attention and development efforts. The application of the polymer carriers characterized with the required kinetics of AMP release (constant time, delayed time, or pulsating release) becomes possible. In this article, we aim to demonstrate some of the needs and current directions in developing polymeric AMP carriers and their potential applications in the biomedical or pharmaceutical fields. 

## 2. Polymeric Systems of Peptides with Antimicrobial Activity

There are many methods for obtaining polymeric AMP systems. AMPs could be immobilized via incorporation into a variety of materials or adsorption onto a variety of surfaces and still retain their ability to bind and kill bacteria [10]. AMPs can be targeted through loading them in nanoparticulate systems with selective delivery capacities. These include dendritic polymers, liposomes, hydrogels, nanospheres, nanocapsules, and carbon nanotubes [43,44]. Peptide encapsulation or adsorption on micro- and nanocarriers has been achieved by various methods like emulsion polymerization, interfacial polymerization, solvent evaporation, salting out, coacervation, combination of sonication and layer by layer technology, solvent displacement, or solvent diffusion *etc.* [45,46].

The release of AMPs from the degradable delivery systems can be governed by several mechanisms: pure peptide diffusion through the polymer matrix, degradation of the polymer (erosion) and influence of the osmotic pressure (Table 1). However, many biodegradable polymer-AMP delivery systems are very complex and drug release is often the result of a combination of several mechanisms [47]. The kinetic release of AMPs can be different and depends on many factors, e.g., molecular mass and polydispersity of polymer, morphology, environment, presence of enzymes, *etc*. For example, the zero-order release profile depicts the constant release of AMPs from the device over time. However, the first-order release profile is typical of diffusion-controlled systems and is characterized by a decreasing release rate with time [47].

**Table 1:** Polymeric carriers of peptides.

Release Mechanism	Polymer Examples
bulk and surface erosion mechanism	copolymers of lactide and glycolide, polylactide, poly(lactic acid), gelatin, copolymers of poly(ethylene glycol) and lactide or glycolide, polyanhydrides, poly(ortho ester)s blends of poly(ethylene glycol) and poly(lactic acid), poly(ethylene glycol)-*b*-poly(propylene glycol)-*b*-poly(ethylene glycol) and poly(lactic acid), poly(vinyl alcohol), and copolymers of lactide and glycolide
diffusion mechanism	hydrogel systems: poly(ethylene glycol), poly(ethylene glycol)-*b*-poly(propylene glycol)-*b*-poly(ethylene glycol), polyvinylpyrrolidone, poly(vinyl alcohol), copylymers of maleic anhydride and alkyl vinyl ether, cellulose, hyaluronic acid derivatives, alginate, collagen, gelatin, albumin, starches, dextrans
osmotic pressure	polyurethanes, polysiloxanes

Incorporation is the most popular method for preparing immobilized AMPs. In [48] the incorporation and release of ponericin G1 (amino acid sequence: GWKDWAKKAGGWLKKKGPG MAKAALKAAMQ) from hydrolytically degradable matrixes (composed of poly(β-amino ester), chondroitin sulfate, alginic acid and dextran sulfate)) was described. The films were effective in inhibition of the growth of *Staphylococcus aureus*, which commonly causes infections. Additionally, the film-coated substrates inhibited *S. aureus* attachment, a necessary step in preventing the formation of biofilms on surfaces.

The mixture of simvastatin hydroxyacid (SIM), parathyroid hormone (1–34) (PTH(1–34)), and the antimicrobial peptide cecropin B (CB) was also incorporated using a complexation polymer system composed of cellulose acetate phthalate (CAP) and Pluronic F-127 (PF-127) (blend ratio, 7:3). It was found that with CAP/PF-127 microspheres loaded with CB and SIM, CB and PTH(1–34), or SIM and PTH(1–34), intermittent release with five distinguishable peaks for each of the two molecules was achieved [49].

Recently, a WLBU2 peptide antibiotic, a 24-aminoacid peptide derived from lentivirus lytic peptide with broad activity against both Gram-positive and Gram-negative bacteria, was incorporated into a bioerodible polymer capable of localized drug delivery [50,51,52]. Furthermore, WLBU2 has been shown to be effective against oral bacteria [53]. The interaction of WLBU2A with bioerodible association polymer comprising of cellulose acetate phthalate (CAP) and Pluronic^®^ F-127 (PF-127) was also examined [44]. The intrinsic antimicrobial activity of CAP/PF-127 and the combined effects of the polymer and WLBU2 were examined using *Streptococcus gordonii*, a species involved in early colonisation of tooth surfaces. It was found that interaction between the WLBU2 and the CAP/PF-127 polymer blend reduced the bactericidal effect of the peptide on the oral bacterium *S. gordonii*, even though the polymer itself is bacteriostatic [44].

AMPs can be incorporated into polymeric films to retard spoilage and increase food preservation times [10]. In polyethylene film, nisin inhibits the growth of *Brochothrix thermosphacta* on beef surfaces at 4 °C for up to 21 days [54]. In polyelectrolyte multilayer films, AMPs (like the defensin from *Anopheles gambiae* mosquitoes) reduces the growth of *Escherichia coli* by about 79% and *Micrococcus luteus* by about 86% in growth assays. Insect defenses are members of a widely distributed family of AMPs (containing from 36 to 46 amino acids) with a typical pattern of six cysteine residues and three disulfide bridges [10,55]. In polyelectrolyte multilayer films on silicon wafers, gramicidin A both prevents the growth of *Enterococcus faecalis* and lyses the microbial cells that do attach [10,56].

AMPs can also be incorporated into resins or brush layers and used as contact-active cationic antimicrobial surfaces [10,57,58]. The model peptide KLAL and magainin-derived peptide MK5E immobilized on resins have antimicrobial activity towards *E. coli* and *Bacillus subtilis* [10,57]. It was found that the immobilized peptides reduce the antimicrobial activity but not the spectrum of activity. Longer spacers between the resin surface and KLAL or MK5E and the chain position of immobilization are more important to antimicrobial activity than the surface density of the peptides [10,57]. Magainin I incorporated into 2-(2-methoxyethoxy)ethyl methacrylate and hydroxyl-terminated poly(ethylene glycol) methacrylate retains antibacterial activity against the food-borne disease-inducing microorganisms *Listeria ivanovii* and *Bacillus cereus* [10,59].

Recently, we found that aliphatic polyesters or poly(ester-carbonate)s are satisfactory carriers for citropin. The biodegradable or bioresorbable polymeric carriers have been obtained by the ring-opening polymerization of ε-caprolactone (CL), *rac*-lactide (*rac*-LA), L-lactide (LLA), glycolide (GLY), and trimethylene carbonate (TMC). The degree of degradation of polyester- and poly(ester-carbonate)s carriers of citropin has been tested *in vitro* under different conditions. The results obtained demonstrate that the homo- and copolymers of CL, *rac*-LA, LLA, GLY and TMC are interesting materials for the controlled release of citropin. Extra experimental details and arguments will be given in our subsequent papers.

Adsorption is the second method for obtaining immobilized AMPs. AMPs can be adsorbed to surfaces with proposed uses as contact-active cationic antimicrobial surfaces [10]. Nisin adsorbed e.g., onto poly(ethylene terephthalate) and rubber surfaces inhibits the growth of *Enterococcus hirae.* Nisaplin—a congener of nisin—adsorbed onto surfaces reduces the attachment of *Listeria monocytogenes* [10,60]. Microbial counts of skim milk in nisin-adsorbed poly(ethylene terephthalate) bottles are significantly lower after 24 days of refrigerated storage [10,60].

Encapsulation is another method for producing polymeric AMPs systems. AMPs (e.g., LL-37) can be encapsulated within silica or titania nanoparticles to create bionanocomposite materials with antimicrobial activity for use as broad-spectrum antifouling materials or cosmetics with antimicrobial and sunscreen properties [10,61]. Lysozyme catalysed the precipitation of silica from tetramethoxysilane to form interconnected nanospheres entrapping the biologically active substances. LL-37 (amino acid sequence: LLGDFFRKSKEKIGKEFKRIVQRIKDFLRNLVPRTES) in silica precipitated from tetraethyl orthosilicate is slowly released and has high antimicrobial activity against *E. coli* and *S. aureus*, low haemolytic activity for erythrocytes and low cytotoxicity against keratinocytes [10,62].

Immobilized AMPs can be also used as biosensors. Cecropin A (amino acid sequence: KWKLFKKIEKVGQNIRDGIIKAGPAVAVVGQATQIAK-NH_2_), magainin I (amino acid sequence: GIGKFLHSAGKFGKAFVGEIMKS), and parasin (amino acid sequence: H-KGRG KQGGKVRAKAKTRSS-OH) immobilized on silanised glass slides with bifunctional N-(-maleimidobutyryloxy) succinimide ester spacers binds *E. coli* O157:H7 and *Salmonella enterica* serovar Typhimurium (detection limits were 0.5–5.0 × 10^5^ colony-forming units (CFU)/mL for *E. coli* and 0.1–5.0 × 10^6^ CFU/mL for *S. enterica* serovar Typhimurium) [10,63,64].

AMPs can be immobilized and their proposed uses include denture materials, which could form preventive biofilms. AMPs immobilized to titanium surfaces may serve to shorten the period of osseointegration of implants and reduce colonization of periodontopathogens on implant surfaces [10,65,66,67]. Immobilised histatin 5 (amino acid sequence: DSHAKRHHGYKRKFHEKHHSHRGY), immobilised conjugated peptides of histatin 5/titanium-binding peptide and lactoferricin/titanium-binding peptide reduces colonisation of *Porphyromonas gingivalis* and enhance the mRNA expression of Runx-2, OPN and ALPase in osteoblastic cells [10,65,66,67].

Polymeric gels can be used as controlled release media for AMP systems. For example, poly(methacrylic acid) (PMAA) ultrathin hydrogel coatings that release antimicrobial agents (AmAs) in response to pH variations have been obtained by Libera *et al*. AmAs included gentamicin and an antibacterial cationic peptide L5. It was found that L5 retained its antibacterial activity toward planktonic *Staphylococcus epidermidis* after released from PMAA hydrogels [68,69,70].

AMPs can be immobilized onto polymeric solid supports either physically (adsorption or “layer-by-layer” assembly) or chemically (covalent bonds) [71]. In the “layer-by-layer” method, AMPs are sandwiched between two polyionic polymers. The diffusion process of the embedded AMPs in the multilayer materials is more complex than diffusion in solutions. Additional factors such as the tortuosity of the diffusion pathway, assembly thickness and peptide-polymer interactions can significantly influence the diffusion process [71,72,73,74]. The multilayer assembly is effective in preventing bacterial growth through controllable and continuous release of AMPs to the interface, but the long-term stability of these assemblies is still largely undocumented [57,71,73,75,76].

## 3. Macromolecular Peptide Conjugates with Antimicrobial Activity

**Figure 1:**
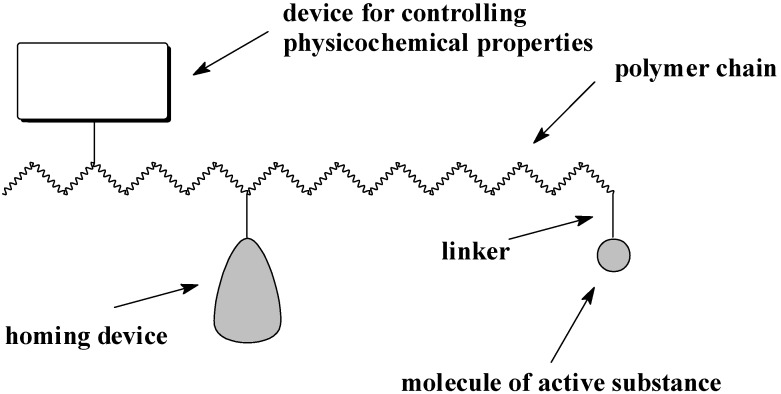
Ringsdorf model of synthetic polymer prodrugs.

**Figure 2:**
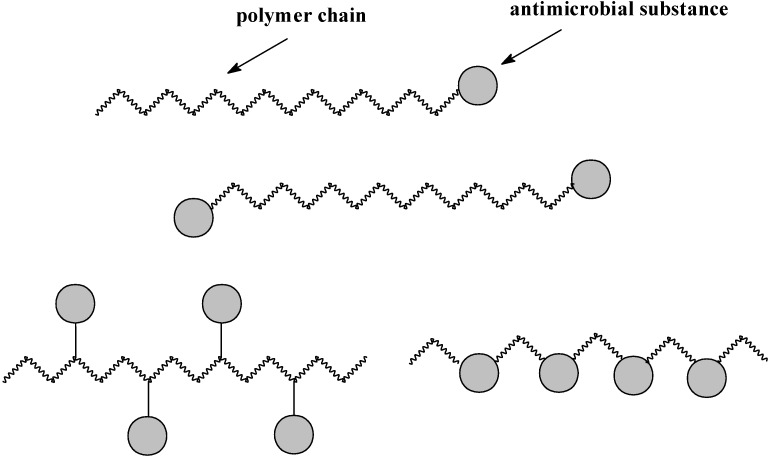
Structure of macromolecular conjugates of active substances.

As mentioned above, AMPs may be also delivered in the form of covalent conjugates with polymers. In 1975, Ringsdorf proposed a model for the rational design of polymeric prodrugs [9,77]. His model is based on the covalent link between the drug and a macromolecular backbone through a labile bond. Figure 1 shows a scheme of the Ringsdorf model, where a biostable or a biodegradable polymer backbone carries three different units. The macromolecule consists of the first region-devices controlling physicochemical properties; the second region in which the drug is linked to the polymer chain, and the third area that incorporates a transport system whose function is to carry the whole polymer to the target cells or site of pharmacological action [77]. In general, the molecule of an antimicrobial substance could be incorporate into polymer chain, might be end-capped or may form a pendant group of the macromolecular chain (Figure 2).

Different strategies for the preparation of AMP-polymer conjugates are known, namely the coupling method (peptide segments are coupled to the preformed polymer blocks using one or multiple reactive sites), the polymerization method (the synthetic polymer block is grown *in situ* from the peptide segment), the inverse conjugation method (the peptide is sequentially assembled on a preformed synthetic polymer), and the macromonomer method (the polymerization of short peptide segments that possess a polymerizable functionality and lead to comb structures). The different methods can be performed using solution-phase chemistry or in a solid-phase supported manner [19,23].

Polymer candidates for AMPs-macromolecular conjugate preparation should fulfil a number of requirements: the availability of suitable functional groups for the covalent coupling with peptides, biocompatibility, biodegradability or a molecular weight below the renal excretion limit [9].

**Figure 3:**
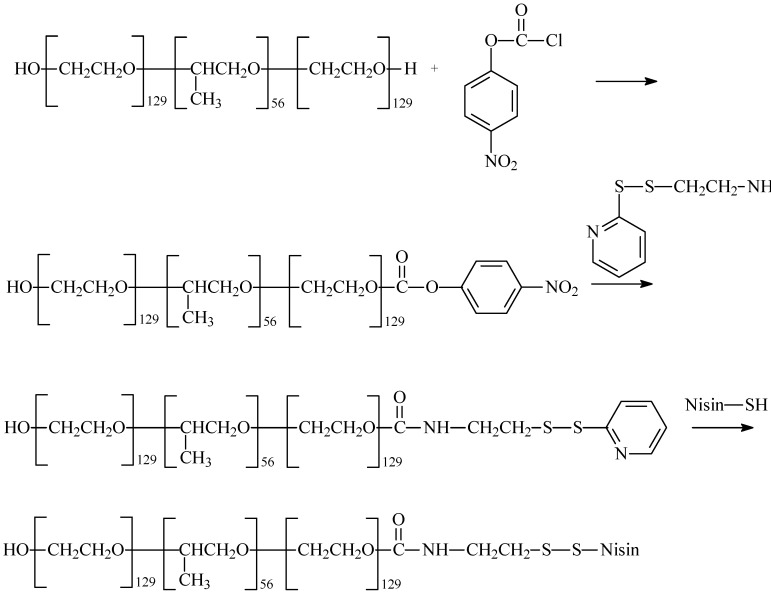
Synthesis scheme of polymeric conjugates of nisin.

Poly(ethylene glycol) (PEO) or copolymers of ethylene glycol and propylene glycol are the most often used substances in the synthesis of macromolecular conjugates of AMPs. PEO-*b*-PPO-*b*-PEO triblock copolymers were covalently linked with nisin (a polycyclic antibacterial peptide with 34 amino acid residues used as a food preservative; in addition, it is a rare example of a “broad-spectrum” bacteriocin effective against many Gram-positive organisms, including lactic acid bacteria, *L. monocytogenes*, *S. aureus*, *B. cereus*, *Clostridium botulinum*, *etc.*) [78]. Thiolated-nisin prepared as mentioned above was reacted with an end-group-activated Pluronic^R^ F108 (*M*_W_ = 14 600 g/mol, HO-(CH_2_CH_2_O)_129_ (CH(CH_3_)CH_2_O))_56_-(CH_2_CH_2_O)_129_H) that had been modified by the conversion of terminal hydroxyl groups of the PEO chains to pyridyl disulfide groups (Figure 3) [78,79]. The antibacterial activity of the modified-nisin copolymer was determined by an agar well diffusion assay against *Pediococcus pentosaceus* bacteria. It was found that the nisin linked to the modified copolymer showed activity similar to that observed in a simply blended sample [8,78].

Brushes of 2-(2-methoxyethoxy)ethyl methacrylate and hydroxy-terminated poly(ethylene glycol) methacrylate statistical copolymer were functionalized with magainin I. The antibacterial activity of these materials was tested against the bacteria *L. ivanovii* and *B. cereus*. The results have indicated a strong biocidal effect, even for the low degree of peptide incorporation [8].

Polystyrene resin beads with surface-grafted poly(ethylene glycol) (PEG) were covalently linked with an antimicrobial peptide with amino sequence HOOC-LKLLKKLLKLLKKL-NH_3_. The AMP was composed of eight lysine and seven leucine (6K8L) residues. The peptide was synthesized by the Fmoc method (solid-phase peptide synthesis SPPS) at the terminal end of the PEG spacer after the PEG had been attached to the PS. The carboxyl end of the first amino acid residue (Leu) was esterified to the free end of the PEG. Coupling of each subsequent amino acid was accomplished using 2-(*H*-benzotriazole-1-yl)-1,1,3,3 tetramethyluronium tetrafluoroborate, 1-hydroxybenzotriazole and *N*-methylmorpholine. The received materials were tested against *B. subtilis*, *E. coli*, *L. monocytogenes*, *Pseudomonas fluorescence*, *Salmonella typhimurium*, *Serratia liquefaciens*, *S. aureus*, and *Kluyveromyces marxianus* yeast, showing an antimicrobial activity directly related to the concentration of the modified PS, pH medium and the exposure time [80].

Synthesis of a triblock bioconjugate with a different arrangement of the blocks was described by Becker [81]. The macromolecular conjugate consisted a terminal tritrpticin (amino acid sequence: VRRFPWWWPFLRR) segment with an amphiphilic copolymer. The tritrpticin-*b*-poly(acrylic acid)-b-poly(styrene) assembled in water into micellar aggregates, mainly driven by the amphiphilicity of the block copolymer. The self-assembly of the triblock macromolecular conjugate led to the positioning of the antimicrobial tritrpticin segment of the micellar surface, making it easily accessible from the water phase. The poly(acrylic acid) segment of the conjugate was used to cross-link the shell of the micellar construct, which stabilizes the functional nanoparticles. The preliminary study of the antimicrobial properties of the received materials was compared to their activity [81]. The minimum inhibitory concentration (MIC) was determined for *S. aureus* and *E. coli*. The tritrpticin was effective against *S. aureus* and *E. coli* at 17 and 33 μg/mL, respectively. In comparison, the micellar aggregates exhibited reduced MIC values of 13 μg/mL for both *S. aureus* and *E. coli* [23,81].

A highly biocompatibile polydimethylsiloxane (PDMS) were used in the synthesis of polymeric conjugates of AMPs. The salivary peptide histatin 5 and two synthetic variants (Dhvar 4 and Dhvar 5) were used to prepare peptide-functionalized PDMS using 4-azido-2,3,5,6-tetrafluorobenzoic acid (AFB) as an inter-linked molecule [8,82]. Polylysine-, polyarginine- and polyhistidine-PDMS surfaces were also prepared. The results have shown that Dhvar 4-functionalized PDMS yielded the highest reduction of the number of *Candida albicans* biofilm cells. It was also found that inhibition of *C. albicans* biofilm formation is highly depended on the nature of the immobilized AMP, its molecular weight, and stereochemistry. For example, poly(D-lysine)-PDMS with molecular weight lower than 15,000 Da and Dhvar 4-PDMS are able to reduce ca. 90% of live cells in a 10^6^ CFU/mL broth culture after 24 h [8,82].

The covalent immobilization of AMPs onto biomaterial surfaces is recommended for health applications as antimicrobial coatings of medical devices [83]. In the covalent coupling, AMPs react chemically with a given surface to form stable antimicrobial coatings [57,58]. The concentration of bound peptides may vary significantly from one surface to another, depending on the density of the reactive groups on the surface (alcohol, aldehyde, amine, carboxylic acid, epoxide, maleimide, isothiocyanate, or thiol surface), in addition to other factors such as coupling conditions and steric hindrance effects [71]. The length of the spacer can be varied from one to several carbon atoms, depending on the significance of spacer length effect on AMP activity. The spacer length is more critical in influencing the activity of bound AMPs than surface concentration. Additionally, the orientation of bound AMP can be controlled through the utilization of suitable chemo-selective coupling reactions enabling detailed study of the effect of peptide orientation on their biological activity [71,84,85].

**Figure 4:**
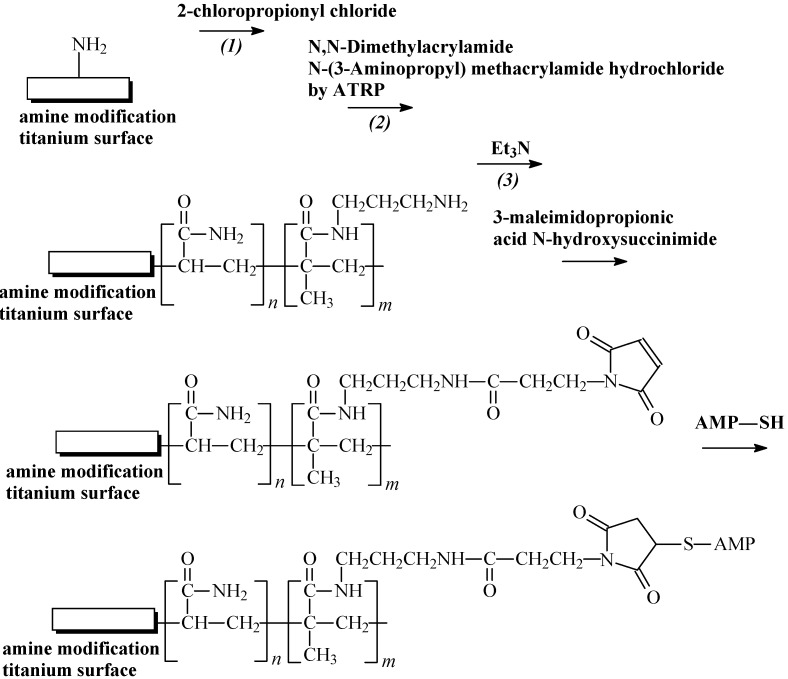
Synthesis of AMPs immobilized copolymer brushes on surfaces.

The development of a specially structured infection-resistant coating for implants based on covalently grafted hydrophilic polymer brushes conjugated with an optimized series of tethered AMPs has been described [86]. Primary amino-functionalized copolymer brushes containing *N*,*N*-dimethylacrylamide and aminopropyl meacrylamide hydrochloride were obtained, using aqueous surface-initiated atom transfer radical polymerization (Figure 4). A titanium deposited silicon wafer was used as a model surface for the optimization of the surface chemistry, and for the determination of the polymerization conditions and polymer brush properties. Tet-213 (amino acid sequence: KRWW KWWRRC), 1010cys (amino acid sequence: IRWRIRVWVRRIC), Tet-20 (amino acid sequence: KRWRIRVRVIRKC), Tet-21 (amino acid sequence: KKWKIVVIKWKKC), Tet-26 (amino acid sequence: WIVVIWRRKRRRC), HH2 (amino acid sequence: VNLRIRVAVIRAC), MXX226 (amino acid sequence: ILRWPWWPWRRKC) were used as AMPs. It was found that the polymer brush tethered AMPs showed an excellent broad spectrum of antimicrobial activity as well as biofilm resistance *in vitro* and it depended on the types of AMPs used [86].

Magainin 2 and the several synthetic amphipathic peptides covalent immobilized onto a polyamide resin retained lethal activity against several Gram-positive and Gram-negative bacteria. It was found that the interaction of magainin 2 with the outer membrane of the bacteria is sufficient for the lethal activity, since the potential peptide penetration depth is very low due to the short spacer (2 or 6 carbon chain linkers) used [58]. The prevention of bacterial colonization and formation of bacterial biofilms on implant surfaces has been a challenge in orthopedic surgery. AMP-eluting coatings on implants are one of the most promising strategies that have been attempted.

## 4. Summary

Polymeric delivery systems for peptides with antimicrobial activity have been described in this review. These systems seem to be an interesting and promising developmental direction for medicine, pharmacy, food technology, *etc.* due to their unique peptide release kinetics. The described systems included types in which the peptide is dispersed throughout the polymer matrix or peptide-polymer conjugates and those where the active substance is covalently bound to the polymer chain. The development of new and effective polymeric systems as well as the preparation and application of specific controlled delivery formulations offers enormous possibilities for the present and the future advanced technology of AMP systems.
